# 617. Laboratory capacities to diagnose and treat Invasive Fungal Infections in Europe: results from an ECMM survey

**DOI:** 10.1093/ofid/ofad500.683

**Published:** 2023-11-27

**Authors:** Jon Salmanton-Garcia, Martin Hoenigl, Jean-Pierre Gangneux, Ester Segal, Oliver A Cornely

**Affiliations:** University Hospital Cologne, Cologne, Germany, Cologne, Nordrhein-Westfalen, Germany; Medical University of Graz, Department of Internal Medicine, Division of Infectious Diseases, Graz, Steiermark, Austria; CHU de Rennes, Rennes, France, Rennes, Bretagne, France; Technion Tel Aviv, Tel Aviv, Tel Aviv, Israel; University of Cologne, Cologne, Germany, Cologne, Nordrhein-Westfalen, Germany

## Abstract

**Background:**

Invasive fungal infections (IFI) are a major threat for patients under immunosuppression or with viral respiratory infections, like influenza or coronavirus disease 2019 (COVID-19). Access to appropriate tools is vital for early diagnosis and clinical management. The European Confederation of Medical Mycology (ECMM) survey on laboratory capacities in Europe aims to decipher the current diagnostic capacity and availability of treatments for IFIs in order to guide health professionals, patients, and policymakers.

**Methods:**

The ECMM IFI diagnostic capacity survey is online accessible at www.clinicalsurveys.net/uc/IFI_management_capacity/. A campaign seeking for mycologist feedback was launched. The survey was disseminated to all European mycologists affiliated with the ECMM and via social media (i.e., LinkedIn or Twitter) and email. Collected variables were a) institution profile, b) perceptions on invasive fungal disease in the respective institution, c) microscopy, d) culture and fungal identification, e) serology, f) antigen detection, g) molecular tests and h) therapeutic drug monitoring.

**Results:**

258 centers from 41 countries have participated in the survey (Figure 1). Germany (n=30), France (n=28), Italy, Spain (n=23, each) and Turkey (n=21) were the origin country of almost half of the respondents. Incidence of IFI was considered as very low or low in 46.9% of the institutions, and moderate in 38.0%. *Candida* spp. (95.0%) and *Aspergillus* spp. (89.9%) were considered the most relevant pathogens. All the institutions had access (on site or outsourced) to cultures (68.2% of which could also perform susceptibility tests in molds and yeasts, 26.4% only in yeasts, 4.7% in none and 0.8% only in molds). Regarding availability of other diagnostic tests, 84.5% could also utilize microscopy, 83.7% antigen detection tests (89.8% of which at least *Aspergillus* galactomannan test), 73.3% molecular tests (mainly PCR), and 62.4% to serology. At least one triazole was available for prescribing in 93.0% of the institutions, whereas at least one echinocandin in 90.3% and liposomal amphotericin B in 80.2%.

**Conclusion:**

Europe is generally well prepared to diagnose and treat invasive fungal infections. However, some institutions miss access to certain diagnostic tools and antifungal drugs.

**Disclosures:**

**Martin Hoenigl, n/a**, Astellas: Grant/Research Support|Euroimmun: Grant/Research Support|F2G: Grant/Research Support|Gilead: Grant/Research Support|Immy: Grant/Research Support|MSD: Grant/Research Support|Mundipharma: Grant/Research Support|Pfizer: Grant/Research Support|Pulmocide: Grant/Research Support|Scynexis: Grant/Research Support **Oliver A. Cornely, MD PhD**, DZIF: Advisor/Consultant|DZIF: Board Member|DZIF: Grant/Research Support|DZIF: Honoraria|DZIF: Stocks/Bonds

